# EHMTI-0142. Features of the headache secondary to unruptured intracranial aneurysm with oculomotor nerve paresis

**DOI:** 10.1186/1129-2377-15-S1-C43

**Published:** 2014-09-18

**Authors:** Y Matsumori, S Fujiwara

**Affiliations:** 1 Headache Medicine, Kohnan Hospital, Sendai, Japan; 2 Neurosurgery, Kohnan Hospital, Sendai, Japan

## Introduction

The symptoms of unruptured intracranial aneurysms (UIA) may be shown the oculomotor nerve paresis (ONP). Those cases should be treated immediately as an impending rupture.

## Aims

We evaluated the headache secondary to UIA with ONP.

## Methods

Seventeen cases of UIA with ONP were examined. All cases were treated surgically to prevent the rupture by 6 clipping, 9 IVR, 1 bypass-parent artery occlusion and 1 bypass+IVR. The localizations of aneurysms were as follows, 12 IC-Pcom, 2 IC-Acho, 2 BA-SCA and 1 BA bifurcation, including 2 large IC-PC and 1 giant BA-SCA.

## Results

Except for one case, no one had an episodic headache history. Thirteen cases had a headache besides the all preceded ONP. Ten of whom experienced a pain around orbital region in 9 IC-Pcom and 1 IC-Acho, two of whom had dull headache in 1 giant BA-SCA and 1 BA bifurcation and one of whom had details unknown headache in 1 BA-SCA. Four cases had no episode of a headache in 1 IC-Pcom, 2 large IC-Pcom and 1 IC-Acho. Although it took 11.2 days from onset of a headache to ONP, it was only 6.1 days especially in the cases with an orbital pain. The typical characteristics of an orbital pain were sharp pain to disturb a sleep, no effect on analgesic and sudden onset. In 6 cases performed clipping, very thinner aneurysmal walls about to rupture were observed without exceptions.

## Conclusions

In cases of IC-Pcom and IC-Acho with a diameter of 12 mm less, an orbital pain trended to precede ONP. Since no headache was observed in 2 large IC-Pcom seemed to grow gradually, the pain especially in orbital region may relate in the stimulus to just local tent edge due to the abrupt growth of aneurysm and in the vascular pain itself by aneurysmal wall dilatation. There was a possibility that the pain-generating mechanism of an orbital pain in this series may be similar with it of cluster headache presenting a typical orbital pain, which is considered the origin of pain in internal carotid artery near the cavernous sinus.

No conflict of interest.

